# Listening to COVID-19 survivors: what they need after early discharge from hospital - a qualitative study

**DOI:** 10.1080/17482631.2022.2030001

**Published:** 2022-01-26

**Authors:** Min Guo, Min Kong, Wenxin Shi, Man Wang, Haixia Yang

**Affiliations:** a School of Nursing, Xi’an Jiaotong University Health Science Center, Xi’an, Shaanxi, China; b Zhejiang University School of Medicine, Hangzhou, Zhejiang, China; c Department of Traditional Chinese Medicine The Second Affiliated Hospital of Xi'an Jiaotong University, Xi’an, Shaanxi, China

**Keywords:** COVID-19, pandemic, survivors, post-discharge experience, qualitative study

## Abstract

**Purpose:**

This study aims to explore the post-discharge experience and needs of COVID-19 survivors.

**Methods:**

A qualitative study was conducted. A total of 16 post-discharge COVID-19 patients aged 31–68 years were recruited. The semi-structured interviews were conducted by telephone one month after discharge and were analysed by Colaizzi’s 7-step method.

**Results:**

The post-discharge experience of COVID-19 patients were classified into four categories and ten subcategories. Category I: physiological problems consisted of physical sequelae (such as fatigue, shortness of breath, sleep disorder, chest pain) and a lack of physical rehabilitation guidance. Category II: psychological issues included anxiety, depression, fear, and psychological trauma. Category III: social issues included social stigma and financial stress. Category IV: positive experience and change included gratitude to social support and cherishing life and family.

**Conclusions:**

COVID-19 survivors urgently need guidance for physical rehabilitation and psychological growth, social support provisions, and protection from social stigma. The experience of COVID-19 survivors is significant for planning healthcare management systems and guiding public health prevention efforts.

## Background

Coronavirus disease 2019 (COVID-19) is a highly contagious disease caused by severe acute respiratory syndrome coronavirus 2 (SARS-CoV-2; Ahn et al., 2020). In China, COVID-19 was first identified in December 2019 in Wuhan and had quickly spread, and the number of infections and deaths caused by COVID-19 continues to rise to this day. While the epidemiology, clinical characteristics, and treatment of COVID-19 patients have been extensively studied and clearly described, the post-discharge problems faced by COVID-19 survivors remain unknown. Understanding the course of patients’ recovery from COVID-19 is significant for planning healthcare management systems and guiding public health prevention efforts.

Wuhan, as the epicentre of COVID-19 in China, was hard-hit in the early stages. Although the Chinese government implemented a series of measures to contain the rapid spread of this virus, the medical system in Wuhan collapsed in the early stage of the outbreak. The prevention and treatment of COVID-19 were still in the exploration stage, and the patients’ health deteriorated sharply. Therefore, patients experiencing COVID-19 at the early stage of the outbreak suffered more than those who contracted this disease later on. Hence, exploring the experience and needs of COVID-19 survivors after early discharge from the hospital in Wuhan may provide evidence for intervention in a population affected by COVID-19.

Clinical recovery is not equal to full recovery as a result of the long-term consequences of COVID-19. Beyond stresses inherent in the illness itself, various factors such as disruption of daily routine, inadequate information from public health officials, and stigmatization raise concerns about what COVID-19 survivors experience after discharge. Past studies indicate that an infectious disease impacts the well-being of the individual and the community, and these impacts can persist long after the outbreak (Lau et al., 2008). Based on the rapidly evolving COVID-19 pandemic and the psychosocial effects during past outbreaks of infectious diseases, it is critical to pay more attention to the experience of COVID-19 survivors to understand how we can better support them in dealing with the current situation. Most recent studies have used quantitative approaches to understand the physiological consequences experienced by COVID-19 survivors (Bellan et al., 2021; Halpin et al., 2021; Huang et al., 2021; Zhao et al., 2020). Only a few studies have been conducted to comprehensively understand the needs of the survivors after early discharge from the hospital using qualitative methods. Therefore, our research aims to describe the experience of these COVID-19 survivors to support them effectively. Summarizing the post-discharge experience of COVID-19 survivors in Wuhan has an important practical significance for the medical staff, working together with other care providers, to cope with the impact of COVID-19.

## Methods

### Design and participants

We used a phenomenological method and conducted a qualitative study of post-discharge COVID-19 survivors from Wuhan in April 2020. Using a purposive sampling method, 16 survivors were recruited from the Sino-French New City Branch of Tongji Hospital in Wuhan. The study included individuals who (1) were confirmed to have COVID-19, (2) demonstrated good verbal expression, and (3) were willing to participate in this study. The sample size was determined when data saturation was reached, i.e., after 14 interviews, similar themes started emerging. Moreover, two participants were interviewed to increase the validity of data saturation.

### Data collection

The interviewers were two nurses, one of whom was appointed to the Sino-French New City Branch of Tongji Hospital to care for COVID-19 survivors. Both the interviewers underwent unified training before conducting the interview to ensure consistent methods and procedure. The nursing manager of this hospital provided the interviewer with contact information for survivors who met the inclusion criteria. The discharged survivors were contacted by telephone. The purpose and procedures of the study were introduced to the participants in advance, and interviews were performed about one month after discharge via telephone or WeChat. The interview guide (see, Appendix) was developed by all the team members according to the research aims and relevant literature. Two pilot interviews were conducted to identify any unclear questions. The one-on-one interviews were conducted by telephone due to the pandemic. Prior to the interview, the interviewer ensured that the participant was in a private and comfortable environment. Participants’ age, gender, comorbid conditions, clinical classification, etc., were gathered from medical records. COVID-19 clinical classification was determined as mild, moderate, severe, and critical according to the “Guidelines for the diagnosis and treatment of COVID-19” released by the National Health Commission (NHC) of the People’s Republic of China (Xu et al., 2020). Each interview lasted approximately 30–60 minutes.

### Data analysis

Within 24 hours of each interview, the recording was transcribed verbatim. The NVivo 11 statistics software and Colaizzi’s phenomenological analysis method were used for data analysis (Colaizzi, 1978). MG and HY read the transcripts carefully to familiarize themselves with the data and extracted significant statements related to the survivors’ post-discharge experience. They identified meaning units from these significant statements and assigned codes. Themes and subthemes were then obtained from the codes. MG and HY completed the above steps independently, then conducted consistency checks and discussions. When interpretations differed, discussions were carried out with all team researchers, and a professor with qualitative research experience was consulted to reach a consensus. MG, HY, and MK formulated a detailed description of the post-discharge experience of the survivors. The findings were returned to the participants for verification. Table I is an example of the theme coding.

**Table I.** Example of the coding process

Quote	Meaning units	Codes	Subcategory	Category
*“I felt isolated by my relatives. After my discharge, my mother-in-law did not allow me to self-quarantine at home because there were two children there. She told me to go to my mother’s home. However, my younger sister also did not let me live there. I was really sad. Everyone in the community was afraid of me.”* *“Relatives and friends did not want to be close to us and said that they should keep their distance from people who had been infected with the virus. They said that people will always carry the virus once infected. They said that they feared they could get infected from us.”*	Feeling isolated by relatives and friends; Feeling sad; Feared by everyone in the community; Others kept the distance from them; Being thought to carry the virus still even after discharge; Fear of being infected from survivors	Isolation, sadness, labelling and prejudice, afraid	Social stigma	Social problems

### Trustworthiness

The trustworthiness of the data was confirmed based on the credibility, transferability, dependability, and confirmability in the following strategies (Lincoln & Guba, 1985). First, two different researchers independently analysed the data by applying the Colaizzi’s analysis procedures systematically. If their interpretations differed, they discussed them until the most suitable interpretation was found. All transcripts of the interviews and the findings were sent to the participants for feedback. In addition, the purposive sampling method and a thorough description of the participants and the research process were used to ensure transferability. Results were then reviewed by researchers not involved in the initial analysis. We employed strategies to achieve bracketing in this research and asked non-leading questions to the participants about their experience throughout the semi-structured interview. We kept a conscious and open-minded attitude and wrote down our thoughts and feelings to avoid being guided by these pre-suppositions in the process of data analysis. The findings were also shared with the participants to verify whether their answers had been distorted and filtered.

### Ethics

This study was approved by the Xi’an Jiaotong University Health Science Center (Ref: 2020–1331). All participants provided oral consent. We promised that anonymity would be protected, although the interviews would be audio-recorded, and participants were allowed to withdraw consent at any time without consequences. Only the researchers had access to the audio tapes and transcripts. Confidentiality was assured by using numbers instead of names (e.g., H1, H2, etc.) and removing identifying information from the transcripts.

## Results

This study included 16 participants: 6 males and 10 females aged 31–68 years. Six survivors had experienced a severe case of COVID-19, four survivors had a moderate case, and six survivors had a mild case. Eight survivors had comorbid conditions. Three survivors lived alone after discharge, seven survivors lived with a spouse, and six survivors lived with family. Most survivors had one child. Table II outlines the characteristics of the participants. The interview revealed that although each respondent had subjective feelings about their experience with COVID-19, they also experienced many shared emotions. The results included four categories and ten subcategories (Table III).

**Table II.** The characteristics of participants

Participants	Clinical Classification	Age	Gender	Comorbid conditions	Living condition	Offspring
H1	Severe	55	Female	No	Living with family	One child (age≥18)
H2	Moderate	55	Female	Hypertension	Living with a spouse	One child (age≥18)
H3	Moderate	38	Male	Diabetes	Living with a spouse	One child (age<18)
H4	Mild	37	Female	Hypertension	Living with a spouse	One child (age<18)
H5	Moderate	68	Female	Diabetes, hypertension	Living with family	One child (age≥18)
H6	Mild	35	Female	No	Living with family	One child (age<18)
H7	Severe	48	Male	Diabetes, hypertension	Living alone	One child (age≥18)
H8	Severe	39	Male	No	Living with family	One child (age<18)
H9	Severe	57	Female	No	Living alone	One child (age≥18)
H10	Severe	63	Male	No	Living with family	One child (age≥18)
H11	Mild	50	Male	No	Living alone	No child
H12	Severe	50	Female	Hypoglycaemia, hypotension	Living with family	One child (agec18)
H13	Mild	31	Female	Pregnant women	Living with a spouse	Pregnancy
H14	Severe	44	Male	Diabetes	Living with a spouse	Two children (age<18)
H15	Mild	43	Female	No	Living with a spouse	Two children (age<18)
H16	Mild	52	Female	Kidney stone, COPD	Living with a spouse	Two children (age≥18)

**Table III.** Summary of categories and subcategories of survivors’ experience

Categories	Subcategories
I. Physiological problems	Physical sequelae Lack of guidance for physical rehabilitation
II. Psychological problems	Depression Anxiety Concerns about the prognosis of the disease Concerns about transmission to family members despite recovery Fear Psychological trauma
III. Social problems	Social stigma Financial stress
IV. Positive experience and change	Gratitude to social support Cherishing life and family

### Category I. Physiological problems

Survivors had some physical sequelae, including fatigue, shortness of breath, sleep disorders, poor appetite, chest pain, and dry cough. After discharge, they were not equipped with medical consultation channels or adequate guidance for rehabilitation.

#### Physical sequelae

Most respondents reported fatigue, sleep disorders, and poor appetite, and patients who had severe COVID-19 infection reported shortness of breath, chest pain, and dry cough.

*H4 (Female, 37): I used to sleep very well. However, after my father-in-law passed away because of COVID-19, I became a light sleeper who could be awakened by slight movements.*

*H12 (Female, 50): I was told that my lungs have recovered after the tests, but I still have a dry cough, chest tightness, and chest pain. I also have shortness of breath and require oxygen therapy.*

#### Lack of guidance for physical rehabilitation

Survivors stated that few healthcare workers contacted them after they were discharged from the hospital. They wanted to know the cause of their residual symptoms and when they might recover, but did not know how to contact the healthcare staff. Participants were worried about COVID-19 reinfection; however, there was no medical staff available to assess chest CT images and test nucleic acids and antibodies regularly. They did not know what to do other than exercise and consume nutritious foods.

*H12 (Female, 50): I have no medical staff to consult regarding my physical symptoms after discharge. I feel as if I was abandoned. No one is going to take care of me once I am discharged.*

*H14 (Male, 44): I still have a lot of pulmonary fibrosis. Is fibrosis of the lungs irreversible?*

*H16 (Female, 52): I have no one to consult after discharge. I was looking forward to communicating with doctors. My CT scan results have not been good, and this still concerns me. I have no alternative but to exercise more.*

### Category II. Psychological problems

A considerable proportion of survivors still had varying degrees of psychological distress after hospital discharge, including depression, anxiety, fear, and psychological trauma.

#### Depression

Participants reported that Wuhan’s healthcare system was chaotic in the early days of the outbreak, with no standardized or regulated diagnostic and treatment procedures for COVID-19 and limited hospital beds. These conditions delayed the hospital’s admission of COVID-19-infected patients and their family members. Six participants stated they had family members, who were also diagnosed with COVID-19, and three of them lost loved ones, making them feel dejected. Some survivors reported that they had done their best for self-protection and quarantine by wearing masks regularly and social distancing from others; however, they were unfortunate to be infected. They felt depressed and betrayed about what happened to them. These mental health challenges existed during and beyond hospitalization.

*H9 (Female, 57): I contracted COVID-19 first, and then my husband got infected because he accompanied me to the hospital. However, he passed away in just a few days. I feel very guilty with regard to him, and I am deeply depressed* (crying).

*H12 (Female, 50): I took things very seriously and did not go out much. I felt that there were many people who were less healthy than me. However, they did not contract COVID-19, whereas I got infected, unfortunately.*

#### Anxiety

Almost all survivors experienced a certain degree of anxiety before and after discharge. According to the participants, the main reasons for their anxiety after leaving the hospital included residual physical symptoms, an uncertain prognosis, and fear of family members being infected.

### Concerns about the prognosis of the disease

Concerns about the prognosis of the disease was one of the main causes of anxiety. Survivors were worried about the potential and irreversible damage caused by COVID-19 to their bodies or organs or a relapse. Insufficient evidence about the prognosis of COVID-19 infections, survivors’ residual symptoms, and exaggerated information from social media contribute to survivors’ concern about the COVID-19 prognosis.

*H1 (Female, 55): I am very anxious and afraid of sequelae.*

*H9 (Female, 57): Will I fully recover from this disease? Some people say that this disease cannot be cured and that the virus remains in the body forever after contraction.*

*H14 (Male, 44): I have already undergone a 30-day self-quarantine at home, but I still do not dare to go out. I am afraid of getting infected again.*

### Concerns about transmission to family members despite recovery

Although most respondents had adopted disinfection and self-isolation measures following hospital discharge, they still worry that their family members may be infected by the virus in their bodies.

*H3 (Male, 38): My wife and I contracted COVID-19. Our child was fine. The test results were all normal. However, children do not have immune systems as strong as those of adults. I am afraid that our child could get infected.*

*H13 (Female, 31): I have been worried about the baby. Will my baby get infected when he/she is born? Do we need to quarantine the newborn then? I do not have much access to this kind of knowledge.*

#### Fear

While the Internet allows ample access to information, the uncontrolled bombarding of online content makes it difficult for survivors to distinguish between correct and incorrect information. Compared to previous outbreaks, an overabundance of incorrect information and fake news about COVID-19 has escalated public fear. Respondents expressed hope to obtain disease information from the medical staff rather than from the Internet, believing that healthcare providers have systematic expertise, rich clinical experience, and the most authoritative information.

*H8 (Male, 39): People have no unified understanding of COVID-19, and experts are still researching and exploring. No one will have a clear idea until we have the vaccine. Media reports were inconsistent, with some even saying that the disease would affect the reproductive system.*

*H13 (Female, 31): I am still worried after hospital discharge, feeling as if the virus is different from what we had previously understood. The news presents different opinions every day. I feel that this disease is more mysterious and more contagious than the diseases that we have faced before.*

#### Psychological trauma

Participants in this study were traumatized by their experience as COVID-19 patients. They reported distressing and painful memories of the traumatic event, the panic of possible death, and the fear of relapse. Their psychological trauma was exacerbated by their near-death experience and the fact that they had witnessed many patients die from this infection.

*H8 (Male, 39): I was helpless when the medical system crashed in Wuhan. I was totally incapable of taking care of myself when I was severely ill. This dramatic deterioration left me with no alternatives. I remember the emergency resuscitation scene of the patient lying next to me at two o’clock in the morning, when the medical team was helpless. They could do nothing but provide oxygen and resuscitation medicines to the patient. Finally, when his injections ran out, he died after raising his hands as if he was going to grab something.*

*H9 (Female, 57): I am afraid that I will die if I have a relapse. I was rescued from death when I was first infected. I am very scared.*

### Category III. Social problems: social stigma and financial stress

#### Social stigma

COVID-19 infection not only affected the patients but also their relationships with other people and with society. There is a general fear of infection among people that makes them intentionally avoid COVID-19 survivors. Respondents reported that they were rejected, isolated, and treated with fear and suspicion by people around them. Some respondents concealed the fact that they had the disease because they were afraid of discrimination.

*H8 (Male, 39): At the moment, many people discriminate against me. They seem to confuse asymptomatic infected cases with recovered ones, and consider me to be the former.*

*H9 (Female, 57): Following my self-quarantine, I wanted to visit my mother. However, my older sister told me not to go, if possible, which made me sad. I dare not say to my friends that I had the disease.*

*H10 (Male, 63): I felt isolated by my relatives. After my discharge, my mother-in-law did not allow me to self-quarantine at home because two children were there. She told me to go to my mother’s home. However, my younger sister also did not let me live there. I was really sad. Everyone in the community was afraid of me.*

*H15 (Female, 43): Relatives and friends did not want to be close to us and said that they should keep their distance from people who had been infected with the virus. They said that people will always carry the virus once infected. They said that they feared they could get infected from us.*

#### Financial stress

The government reimbursed the medical expenses of COVID-19 patients, which solved part of their economic pressure. However, because of the implementation of COVID-19 prevention and control policies, the survivors were not allowed to return to work immediately after being discharged from the hospital, nor were they offered sick pay, and their family members were quarantined at home, generating financial crisis for the entire family. Some participants reported that they could not return to work because of residual physical symptoms and social discrimination. Particularly, if survivors had comorbidities or were the main income provider in the family with the responsibility of supporting older adults and children, the financial pressure is even more obvious.

*H7 (Male, 48): I have not been working, but my living expenses are still there. In addition, because I have diabetes and high blood pressure, I take medicines all year-round, some of which are not covered by the national medical insurance. This is a huge financial stress for me.*

*H9 (Female, 57): I am unemployed, and my husband died of COVID-19. I live alone and have to pay the mortgage on my own. What should I do? I get sad when I think about it.*

*H15 (Female, 43): My husband and I got infected. We work for private companies, and we cannot take paid leaves. Moreover, we have children and the elderly to look after. I felt that our family has completely collapsed.*

### Category IV. Positive experience and change

#### Gratitude to social support

In the early stages of the outbreak, when Wuhan suffered from a shortage of medical staff, medical teams were dispatched from other provinces, bringing hope to patients in Wuhan. Almost all respondents expressed sincere appreciation to these medical teams and gratitude to the Chinese government for putting forward the principle of admitting and treating everyone and offering free treatment to all patients, which reduced their financial stress. Respondents claimed that family companionship and care played an important role in overcoming the disease.

*H10 (Male, 63): Thanks to the government’s economic strength and financial support, our medical treatment expenses have been fully reimbursed. Without this support, treatment would not have been affordable to an ordinary family, especially a family like mine with three COVID-19 patients.*

*H8 (Male, 39): I was desperate when I was severely ill. The nurse’s patience increased my confidence in overcoming the disease.*

*H11 (Male, 50): The relationship between doctors and patients used to be quite tense. Previously, I was biased about them. However, I think that the public will thank them with sincere gratitude and reconsider the relationship between doctors and patients after the pandemic.*

*H12 (Female, 50): My husband and son did not avoid me. They took care of me with patience and encouraged me every day.*

*H13 (Female, 31): My husband encouraged me every day. When there were not enough hospital beds, he tried his best to find someone to help me.*

#### Cherishing life and family

Survivors experienced intense physical and mental reactions in struggling with the disease and gradually gained some form of benefit from their experience. This pandemic made respondents realize that they had overlooked health issues in the past and that life is fragile. Therefore, they will cherish life, pay more attention to health, and spend more time with family.

*H11 (Male, 50): From now on, I will strengthen self-protection, improve my lifestyle, increase exercise frequency, and improve immunity. Without good health, the disease can take you away, no matter how rich or famous you are.*

*H12 (Female, 50): I rarely exercised in the past, and I regretted it when I contracted the disease. Thus, now I spend at least one hour a day taking deep breaths or doing aerobics, even if it makes me uncomfortable.*

*H13 (Female, 31): In the future, I will arrange my timetable reasonably, cherish the time spent with family, and do really meaningful things.*

## Discussion

This study found that COVID-19 survivors experienced physical, psychological, and social problems, which fits the framework of the biopsychosocial model developed by George L. Engel (1977). This model focuses on the biological, psychological, and social factors and their complex interactions in understanding health, illness, and healthcare delivery. This study showed that the needs of COVID-19 survivors were not met post-discharge, highlighting the importance of continuous care.

This study found that patients recovering from COVID-19 have residual physical symptoms, including fatigue, shortness of breath, sleep disorders, poor appetite, chest pain, and dry cough. These symptoms may be related to psychological stress or physical damage that has not been fully repaired due to the infection. This is in keeping with a recent quantitative study investigating the symptoms in survivors of COVID-19, highlighting that survivors experienced fatigue, breathlessness, psychological distress, cough, and loss of appetite after discharge (Halpin et al., 2021). Both studies in the UK and Wuhan showed fatigue and shortness of breath were the most common symptoms among COVID-19 survivors (Halpin et al., 2021; Huang et al., 2021). In this study, 10 of the participants had a moderate or higher degree of disease, and half of them had comorbidities, which may be one of the reasons for their poor post-discharge experience. Survivors with a severe degree of the disease during hospitalization or coexisting diseases have increased risks of more symptoms after discharge from hospitals (Zhang et al., 2021). A systematic review reported that respiratory compromise, reduced exercise tolerance, and reduced quality of life were crucial issues in SARS and MERS survivors, and these can persist up to 12 months after discharge (Ahmed et al., 2020). As such, COVID-19 survivors need medical consulting channels or rehabilitation guidelines after hospital discharge. Therefore, it is necessary to identify the symptoms and implement rehabilitation services to manage these symptoms appropriately and maximize the functional return of survivors.

Survivors reported that few healthcare providers offered counselling or support for their continuing discomfort or prognosis information after discharge from the hospital. Even before the emergence of COVID-19, there was already a shortage of public health specialists. According to the 2020 China Health Statistical Yearbook, public health doctors accounted for barely 6.9% of the total medical practitioners (China Health Statistical Yearbook, 2019). Healthcare providers in China, particularly in Wuhan, were overwhelmed by the issues they were facing. When the outbreak reached its peak, there was an emergency shortage of healthcare resources. The limited number of healthcare workers were focused on treating hospitalized COVID-19 patients and were unable to provide adequate follow-up care. This could be one of the reasons for the lack of effective medical support for survivors’ post-discharge. Humanistic care needs to be given to survivors whether or not they have physical symptoms after discharge. This study also highlighted the problem of the spread of distorted information through social media. Medical staff plays a critical role during a pandemic. In addition to providing treatment, care, close observation, and counselling to patients and survivors, they should also provide infection control education to correct any erroneous knowledge and avoid misunderstanding. Thus, increasing the reserve of the medical workforce should be considered, especially health emergency specialists.

The results of the study also showed that most respondents still had psychological distress after hospital discharge, which is consistent with recent research. Anxiety or depression was reported among 23% of discharged patients with COVID-19 in Wuhan (Huang et al., 2021). A recent study in Northern Italy found that 17.2% of COVID-19 survivors had post-traumatic stress symptoms four months after hospital discharge. In this study, the psychological distress of the respondents originated from many factors, including concerns about the recurrence of disease, physical sequelae, stigma, death of family members caused by COVID-19, concerns about children’s health, and financial stress. Early, continuous, and professional psychological interventions can prevent physical and mental harm. Therefore, attention to the psychological aftermath of severe infectious diseases is warranted, and continuous psychological care and social support should be provided in accordance with the different causes of these negative emotions. In the context that face-to-face psychological intervention is not possible due to COVID-19 prevention and control measures, online intervention is feasible as most patients showed a positive attitude towards online mental health services (Bo et al., 2021).

Stigmatization is one of the main experiences of patients with contagious diseases, which is still evident even when the pandemic is over (Lee et al., 2005; Robertson et al., 2004). A review indicated several reasons for the stigma associated with COVID-19: misinformation, feeling of insecurity, fear of responsibility, administrative malfunction, and lack of trust in treatment (Mahmud & Islam, 2021). In this study, respondents were discriminated against by people who lived in the same community, friends, and even family members. Considering stigma can lead to more severe issues such as impaired physical and psychological health and difficulties controlling the disease outbreak (Liu et al., 2020; Mahmud & Islam, 2021), effective action should be taken to avoid fuelling this stigma. For example, using inclusive language and avoiding stigmatizing terminology when talking about COVID-19, collecting and spreading accurate facts to alleviate stigma caused by insufficient knowledge of disease transmission and treatment, and emphasizing the effectiveness of prevention and treatment measures. Additionally, there is an urgent need to amplify survivors’ voices and emphasize to families and friends that their care and support are crucial to the survivors’ recovery.

The results also found that survivors faced unemployment and accompanying financial stress after discharge from the hospital. It has been proven that unemployment is associated with anxiety, depression, and poor quality of life (Hodgson et al., 2018). Therefore, the unemployment and financial stress that survivors would experience after discharge is a significant social issue. Furthermore, the low-income group experienced more severe depression during the pandemic, possibly because people with low income or unpaid leave feel more likely to be affected by income loss during and after the pandemic than the high-income group (Brooks et al., 2020; Hawryluck et al., 2004). In the post-COVID-19 era, the health and workplace systems should assess the specific situations of survivors, and provide appropriate financial and work policies to those suffering the severe impacts of the COVID-19 pandemic.

During the early stage of the COVID-19 outbreak, a majority of the public had little knowledge about the virus. COVID-19’s high hospitalization and casualty rates, as well as the uncertainty regarding the prognosis, increased survivors’ anxiety and panic. Due to the rapidly increased cases and the shortage of hospital beds for COVID-19, some patients with mild or moderate symptoms had to stay home for isolation and observation (Zhuang et al., 2021). This resulted in some patients not receiving timely treatment, whereby their condition deteriorated rapidly. Moreover, by the time they were admitted to the hospital, the disease had worsened. Severe case survivors experienced higher physical sequelae and psychological trauma after being discharged from the hospital (Zhang et al., 2021). Furthermore, home isolation exposed their family to the disease and prompted high intra-family infection rates (Lei et al., 2020), which exacerbated the survivors’ negative psychological and social experiences.

Participants in this study were those who were infected during the early stage of the COVID-19 outbreak. During this period, Wuhan’s healthcare systems showed flaws in their disaster control and prevention measures, including the inability to detect the virus early, overcrowding in public hospitals, and a severe shortage in personal protective equipment and healthcare personnel. Policymakers and hospitals did not have enough time to modify their responses to the abrupt fluctuations. The Chinese Center for Disease Control and Prevention failed to identify, inspect, and respond to outbreaks, putting disease prevention and control at risk. Many patients went to large public hospitals for treatment due to the antiquated diagnostic facilities and limited competency of physicians in community hospitals. As a result of this predicament, hospitals were overburdened with patients. The COVID-19 pandemic put China’s health emergency system to the test. Although the Chinese government has won its fight against the virus by adopting innovative and professional strategies at the later stage, the severe effects of COVID-19 on survivors at the early stage remain. Inadequate long-term material reserves and health emergency personnel, the capacity of community hospitals, and the ability to recognize and respond to emergency medical situations are all issues that must be addressed.

This study also found some positive experiences and change among survivors. COVID-19 survivors rethink and redefine and cherish their lives more, which is one form of post-traumatic growth (PTG). PTG is manifested in many ways, including a greater appreciation for life, greater personal strength, and more meaningful relationships with others (Tedeschi & Calhoun, 1996). We found that patients who reported satisfying social support tend to report psychological growth, which is in keeping with previous studies (McDonough et al., 2014; Zhou et al., 2021). Some interventions, such as cognitive-behavioural therapy, mindfulness-based meditation intervention, and exposure therapy, have been used to strengthen PTG (Danhauer et al., 2013; Hagenaars & van Minnen, 2010; Hanley et al., 2015). Considering that the occurrence of PTG depends on how the event is processed, rather than the event itself, psychological techniques for improving PTG should be adopted to guide survivors to increase positive psychological change and promote physical and mental rehabilitation.

## Limitations

Despite its valuable outcomes, this study had some limitations. First, this study only interviewed survivors from one hospital in Wuhan and did not include survivors from other hospitals, limiting the generalization of these findings. Second, because it was carried out during the pandemic, this study adopted the telephone interview method. The lack of face-to-face communication may have diminished the respondents’ trust in the researcher and resulted in some bias. However, we used WeChat to establish a relationship with survivors to enhance trust.

## Conclusion

COVID-19 survivors urgently need guidance for physical rehabilitation and psychological growth, social support provisions, and protection from social stigma. The experience of COVID-19 survivors is significant for health system planning and guiding public health prevention efforts.

## Appendix. Interview guide

**Table:**

How has contracting COVID-19 affected you? What are the problems you faced after discharge? What kind of help did you require after discharge? Any other aspects that have not already been discussed or that you would like to expand upon?
